# The best possible self‐intervention as a viable public health tool for the prevention of type 2 diabetes: A reflexive thematic analysis of public experience and engagement

**DOI:** 10.1111/hex.13311

**Published:** 2021-07-13

**Authors:** Benjamin Gibson, Kanayo Umeh, Ian Davies, Lisa Newson

**Affiliations:** ^1^ School of Psychology, Faculty of Health Liverpool John Moores University Liverpool, Merseyside UK; ^2^ School of Sports and Exercise Sciences, Faculty of Science Liverpool John Moores University Liverpool, Merseyside UK

**Keywords:** acceptability, experiences, feasibility, intervention, person‐centred, public health, prevention, psychology, qualitative, thematic analysis

## Abstract

**Background:**

Public health initiatives seek to modify lifestyle behaviours associated with risk (e.g., diet, exercise, and smoking), but underpinning psychological and affective processes must also be considered to maximize success.

**Objective:**

This study aimed to qualitatively assess how participants engaged with and utilized the best possible self (BPS)‐intervention specifically as a type 2 diabetes (T2D) prevention tool.

**Design and Methods:**

Fourteen participants engaged with a tailored BPS intervention. Reflexive thematic analysis analysed accounts of participant's experiences and feasibility of use.

**Results:**

All participants submitted evidence of engagement with the intervention. The analysis considered two main themes: Holistic Health and Control. The analysis highlighted several nuanced ways in which individuals conceptualized their health, set goals, and received affective benefits, offering insights into how people personalized a simple intervention to meet their health needs.

**Conclusions:**

To our knowledge, this is the first study to tailor the BPS intervention as a public health application for the prevention of T2D. The intervention enabled users to identify their best possible selves in a way that encouraged T2D preventive behaviours. We propose that our tailored BPS intervention could be a flexible and brief tool to assist public health efforts in encouraging change to aid T2D prevention.

**Public Contribution:**

The format, language and application of the BPS intervention were adapted in response to a public consultation group that developed a version specifically for application in this study.

## 1 BACKGROUND

The worldwide prevalence of noncommunicable diseases (NCDs), such as type 2 diabetes (T2D), is increasing.^1^, ^2^, ^3^ It is estimated that 5 million people are at high risk of developing T2D in England alone.^4^ Following public health calls for an increased focus on prevention,^5^ the UK government has sought to develop T2D prevention programmes and campaigns^4^ with the understanding that prevention is better than management.^6^ However, public health interventions must target the whole population^7^ and cater for all those at risk,^8^ meaning that the absolute success of current interventions is uncertain. For example, initial analysis of the National Diabetes Prevention Programme (2018) reported that 324,699 people had been identified as at risk of T2D and referred for intervention.^9^ However, less than half of those people referred subsequently attended the initial assessment, and in total, 36% attended one intervention session, with only 19% completing the intervention, thus actively supporting only a minority of individuals identified as ‘at risk’.

Although some risk factors for T2D are nonmodifiable, such as genetics, ethnicity, and age, behavioural and environmental changes to diet, exercise, weight status and other health behaviours (e.g., stopping smoking) can aid prevention efforts.^10^ As such, public health interventions often focus on behavioural change in particular to promote healthy lifestyles.^6^ However, T2D prevention also requires the individual to navigate emotional challenges and dysfunction^11^ and associated health‐related cognitions linked to motivation, beliefs and attitudes,^12^, ^13^ complicating their capacity for behaviour modification.

Positive affect (i.e., positive emotion) supports coping and well‐being by providing the mental space for individuals to develop and invest in necessary psychological, intellectual and social resources that they can later draw on in times of need.^14^ Positive affect plays an especially adaptive role in health, as evidenced by its associations with improved physical and mental health outcomes. For example, positive affect is associated with an increase in physical activity,^15^, ^16^ improvements to eating behaviours^17^ and a lower likelihood of tobacco use.^18^, ^19^ Positive affect may even buffer the effects of stress^20^ and depression.^21^ In those identified to be at high risk of T2D, positive affect is linked to improved glycaemic control^22^ and has been shown to protect against the development of T2D among those with a family history of the disease.^23^

Importantly, positive affect can be facilitated to achieve these effects using aptly named positive psychology interventions.^24^, ^25^ Such interventions have been shown to support behavioural and cognitive change in ways that promote well‐being and overall health.^26^ Positive psychology interventions are typically brief and self‐administered,^27^ and their effects are not limited by mental health status.^28^ Consequently, positive psychology interventions could make for an accessible, alternative public health approach, especially as they offer the potential to reach a large population that would benefit from tailored public health intervention, such as those among the general public who may be at risk of developing T2D because of modifiable factors.

Positive psychology interventions have already been shown to improve lifestyle behaviours with specific patient groups. For example, gratitude and self‐affirmation tasks, combined with increased social support, improved physical activity and increased self‐management behaviours in young adults with type 1 diabetes (T1D).^29^ Similarly, a benefit‐finding task was associated with lower depressive symptoms, higher perceived coping effectiveness, improvements in self‐management behaviours, higher positive affective reactions to stress and superior blood glucose levels in a similar population with T1D.^30^ Meanwhile, an online intervention that taught positive affect skills reduced negative affect (i.e., negative emotions) and perceived stress in adults with T2D.^31^ Similarly, a modified version of the ‘best possible self’‐(BPS) intervention was used to help adults with T1D and T2D set diabetes‐specific goals and was shown to improve perceptions of self‐care.^25^ The evidence from these positive psychological interventions is promising, given that they provide evidence that positive psychological interventions are at least associated with changes to behaviour, cognition and affective processes, especially in relation to diabetes. However, the scope of these positive psychology interventions could be broader and applied to support preventative strategies (especially given that positive affect is more likely to support behavioural and cognitive change the earlier into the illness the intervention is provided^32^).

The BPS intervention^33^ could be used for prevention because it is brief, flexible^34^ and well evidenced across various contexts.^35^ Originally developed as an alternative trauma writing paradigm,^36^ the BPS intervention is a disclosive writing exercise designed to help recipients set goals for a positive, imagined future.^37^ There have been consistent empirical findings demonstrating the efficacy of the BPS intervention not just in promoting positive affect and related constructs such as optimism and well‐being.^38^ There are also associations between the BPS intervention and reductions in depressive symptoms,^39^ pain,^40^, ^41^ other physical illness symptoms^25^ and the number of visits to healthcare centres,^33^, ^42^, ^43^ indicating benefits for physical and mental health among clinical populations and the general public alike.

We proposed that the BPS intervention, which targets psychological support, could be an easily administered and acceptable public health intervention. As a first application of the intervention into the general public domain, we investigated how people engaged with and utilized the ‘Best Possible Self’ intervention when used as a T2D prevention tool.

## 2 METHODS

### 2.1 Design

This exploratory, descriptive study has adopted a qualitative design guided by Braun and Clarke's^44^ Reflexive Thematic Analyses. A qualitative investigation can provide rich data around intervention mechanisms, benefits and engagement^45^ and insights into the contextual circumstances of implementing, delivering and evaluating interventions.^46^ Qualitative research in the context of health not only gives voice to patient experiences,^47^ Pope and Mays^48^ argue that it is a ‘prerequisite for good quantitative research, particularly in areas that have received little previous investigation’ (p. 42). Importantly, there is a precedence in the literature for using qualitative analysis to better understand the workings not only of positive psychology interventions^49^, ^50^ but also the BPS intervention specifically.^51^, ^52^ Reflexive Thematic Analysis (TA)^53^ was applied to identify, analyse and report patterns within our data set. TA was chosen as it is best suited to questions related to people's experiences, views and perceptions and the construction of meaning, which we felt was important for understanding how people engaged with the BPS intervention.^53^

### 2.2 Participants and procedure

The study was advertised to adults aged 18 years and over via social media and promoted via North‐West England community groups and UK university mailing lists between May and June 2018. On the basis of the exclusion criteria, people with an existing diabetes diagnosis (any type) and those with severe mental health conditions were not included in this study. Participants had to be able to read and write in English in this initial investigative study.

**Figure 1:**
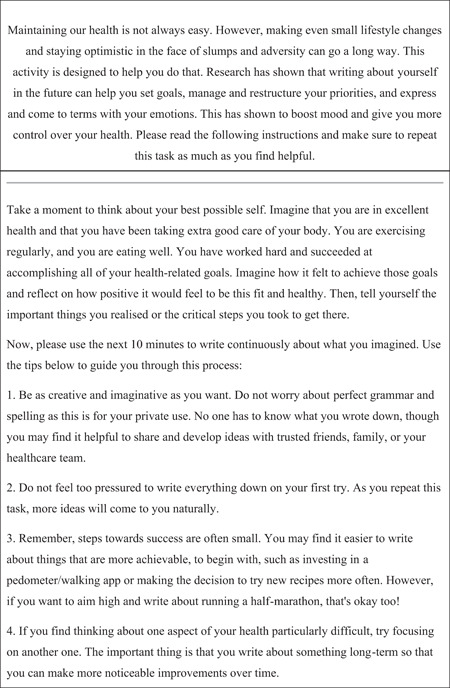
The tailored ‘best possible self’‐instructions

Fourteen people expressed an interest in the study, and the lead author sent further written information via email. Subsequently, all 14 individuals provided full written consent to engage in the research and consent for their submissions to be used for analysis and later publication. The BPS intervention (see Section 2.3 & Figure 1) and user instructions were emailed to the participants. Participants were encouraged to liaise directly (through their preferred communication medium: email, messaging or telephone) with the lead author to seek clarification and ask questions about the intervention before and during the intervention period. At the end of the intervention, a convenience sample of 12 females and 2 males, mean age 30.7 years (SD: 12.7; range: 21–71 years), submitted data.

### 2.3 The intervention

Evidence shows that the BPS intervention needs to be adapted for the user population.^34^ For example, Shapira and Mongrain's^54^ depression–BPS intervention altered the instructions, so recipients were asked to give themselves ‘some sage and compassionate advice from a better future’ (p. 381). The BPS intervention used in the present study was custom‐designed following consultation with members of the general public (*n* = 5), who were recruited through local community health groups.^55^ This consultation consisted of a discussion workshop that utilized the diabetes–BPS^34^, ^56^, ^57^ intervention as a tool for adaptation.^25^ For this study, as explicitly recommended by the public consultation, the language was designed to be sympathetic to the recipient's individual health needs. As suggested, an introduction box was added to the instructions to help people keep health‐related goals in mind when engaging with the intervention. Instructions offered participants a level of flexibility in their approach to stop the task from feeling prescriptive. It was important that the intervention did not dictate which health goals participants had to focus on; participants could decide what aspects of their health they wanted to engage. Unlike the diabetes–BPS intervention,^25^ there was no discussion of clinical outcomes and, conceivably, other populations could use this version of the intervention to tackle other general health‐ and NCD‐related outcomes.

Participants completed the intervention at least once a week (taking approximately 10 min to complete) for a minimum of 4 weeks and then submitted examples of their engagement with the intervention, namely, a written account of their ‘best possible self’. Participants were given time to develop a familiarity with the intervention on a time scale similar to other BPS research.^24^, ^25^, ^34^ Additionally, by allowing participants to engage with the intervention at their leisure (rather than in an experimental setting), it was hoped that participants would feel that they were not writing for anyone but themselves and that accounts would represent a more natural observation of their engagement and represent a true real‐world application.^58^ In addition to the intervention instructions, within the email from the lead author, the participants were asked to consider their engagement (e.g., how/when/where did you complete the intervention), application (how easy/difficult was it to do the intervention) and acceptability (e.g., what did you (dis)like?) of the BPS intervention and to add any such reflective commentaries to their intervention submissions.

### 2.4 Analysis

Informed by the reflective TA literature,^44^ our approach to the analysis was inductive, semantic (people's accounts were mainly taken at face value) and critical realist/essential.^53^ These orientations allowed the team to obtain a rich description of the data set rather than a purely detailed account of one particular aspect. However, the focus of analytical consideration was occasionally shifted to ascertain specific details. For example, it was important to be mindful of the more subtle ways in which the intervention might have affected participants' ideas, goals and behaviours, in which case, this required additional interpretation and discussion within the research team.

Participants posted or email‐returned examples of their intervention accounts, including any additional reflective commentary on their experiences. Reflective commentaries were considered optional data, in addition to the intervention application, and offered the research team insight into the acceptability of the intervention itself. However, to remain in context, reflective commentaries were not extrapolated from the raw data but considered part of the participants (complete) submission. All submissions were classed as raw data, in a similar context to articles that have referred to diary entries^59^, ^60^ or story^61^, ^62^ submissions as data for TA. Participants' accounts were anonymized using a randomly generated pseudonym, and then read and reread by the first author to become familiar with the data.

Initial codes were generated on a line‐by‐line basis, and codes were collated into several candidate themes (Table 1) using traditional pen‐and‐paper methods. These themes were discussed and worked further via the research team. Through open dialogue and ongoing reflections between authors, the themes were amended and continuously checked against the data until only a smaller set of main themes and subthemes remained. The themes were written as part of a series of draft results sections, then scrutinized and reworked between the first, second and last authors until the final version of all themes and subthemes was agreed upon (all authors).

**Table 1:** Initial candidate themes

Candidate themes			
Appearance	Feeling good (quality of life)	Support networks/social aspects of health	How a best possible self affects others
Motivation	Mental health	Specific/quantifiable goals	Existing knowledge
Interconnectedness (how healthy behaviour/mindsets are linked/have beneficial knock‐on effects)	Gaining control over one's health and health behaviours (Identifying what works for you)	A holistic approach to health	Positive feelings generated by considering/achieving goals
BPS as aid to identify/overcome barriers	BPS as means to encourage novel behaviours	Self‐forgiveness	Technology as an aid
Gratitude	The discrepancy between current self and future best possible self (negative)	Other (nonhealth related) goals	Long‐term goals/future expectancies

*Note*: Candidate themes would be merged or otherwise broken down and reworked into larger main themes and subthemes.

Abbreviation: BPS, best possible self.

### 2.5 The research team and methodological rigour

To ensure quality, this study and the writing of this article have, when applicable, applied the Revised Standards for Quality Improvement Reporting Excellence (SQUIRE 2.0) guidelines^63^ and the Consolidated Criteria for Reporting Qualitative Research (COREQ) checklist.^64^ We acknowledge that using Reflective TA, the analysis and subsequent themes were influenced by the research team's subjective interpretations of the data. However, throughout the analytical process, researcher reflexivity and audited discussions^65^ occurred between authors throughout the data collection, analysis and write‐up to ensure rigour in the quality of qualitative analysis conducted.^66^ Researcher triangulation^67^ was conducted through a diverse research team, which promoted objectivity between the researcher's position and the analysis. The lead author was a male, early career researcher with specific interests in intervention development and patient experiences. The second author was a male Senior Lecturer and Chartered Psychologist, with mixed‐methods research expertise and interests across health and technology. The third author was a male Reader in Nutritional Science, with interests in diabetes, nutrition and cardiometabolic risk. The fourth author was a female Reader in Applied Health Psychology and a Registered Health Psychologist with expertise in qualitative methodology, public health and diabetes.

Direct quotes from a range of participants, which we felt would be transparent in context,^66^ acted as evidence to support commentary.^68^ The authors confirm that the raw data examples supporting the findings of this study are available within the article. Due to the nature of this qualitative research, in line with legal and ethical processes, participants of this study did not agree for their full transcripts to be shared publicly, so supporting data beyond the sample quotation extracts are not feasible.

## 3 RESULTS

### 3.1 Overview

**Figure 2:**
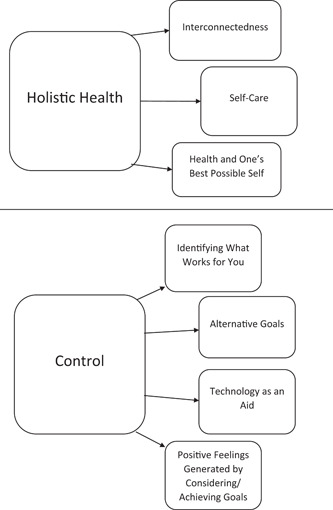
Thematic Map. Data were split into two distinct main themes ('Holistic Health' and 'Control'), which were comprised of three and four subthemes, respectively

The data were developed into two main themes, which summarized their experiences, sense of meaning and reflections of use and value. The first theme, Holistic Health, was informed and influenced by three subthemes: (1) A sense of interconnectedness, (2) Forgiveness and self‐care and (3) Social aspects of health and one's best possible self. The second theme, Control, was influenced by four subthemes: (1) Identifying what works for you, (2) Alternative goals, (3) Technology as an aid and (4) Positive feelings generated by considering/achieving goals. Overall, Holistic Health highlighted how participants thought about their future health in a way that went well beyond their physical needs, while Control emphasized the myriad of ways in which participants used the intervention to think about and challenge themselves to become their ‘best possible self’. See Figure 2 for a simple visual representation of themes.

### 3.2 Holistic health

Participants defined their health broadly, and they demonstrated a frequent acknowledgement of, as well as a desire to, increase their physical, mental and social well‐being. There was an awareness that although physical health behaviours such as diet and exercise were important, aiming to achieve the health of one's best possible self would require a nuanced approach.

#### 3.2.1 A sense of interconnectedness

Participants perceived different aspects of their health as complementary or otherwise linked, which led participants to gain a broader perspective of their health and their goals. For example, several participants made connections between their physical and mental health, which led to an understanding that mental health goals could be used to support physical health goals and vice versa.

‘This highlighted how much I needed to prioritise my emotional health at the moment in order achieve the physical goals I want to achieve’ (6C)

‘The exercise I do in my ideal self include a variety of sports (running, cycling, yoga, climbing, swimming) that improve my fitness in different ways and relax my mind’ (13V)

There was evidence that developing such associations between behaviours even helped goals become easier or more enjoyable.

‘I noticed that exercising makes me eat healthier too. I genuinely crave for fresh fruits and vegetables’ (5D)

#### 3.2.2 Forgiveness and self‐care

Achieving health goals was described by one participant as ‘an ongoing journey’ (4N), and for many participants, this meant taking care of themselves along the way. For 4N, this meant being honest with themselves and celebrating small victories. For others, patience and an ability to persevere despite perceived setbacks were important.

‘This isn't something that happens overnight. You can't eat whatever you want to anymore and it not be an issue; I have to look after my body, we'll be together for a while (hopefully)’ (8E)

For the individual's mental health, in particular, having a ‘structure’ (7B) and being in a position where one could ‘be there for [one's] self’ (1A) were also ways to ensure that visions for a best possible self could be realized.

#### 3.2.3 Social aspects of health and one's best possible self

Personal relationships (friends, family, romantic and acquaintance) were central to informing the participants' BPS accounts. Some of these participants envisioned a more sociable future, either as a goal in itself or as a result of meeting other health goals.

‘I want to become more confident in talking to people, especially strangers, and making myself go up to someone at an event or messaging people more often, so I am not feeling so alone’ (7B)

Others saw their existing support networks as vital for the completion of their newly created health goals; as one participant put it: ‘my best possible self is impacted by other people around me’ (14M). Some individuals suggested that they could not become the best versions of themselves without considering, and even giving back to, others. One participant used the intervention as an opportunity to reflect upon their existing family relationships and seemingly generate a sense of gratitude as a consequence.

‘Still, I am most fortunate in that I have a good marriage, a lovely home, enough money to live on, two super children who are doing well in their careers and one 15‐year‐old grandson who I adore but is a typical teenager at the moment’ (10J)

### 3.3 Control

Goal setting is central to the BPS intervention. This theme highlights how participants were able to take control of their future by making positive decisions in the present.

#### 3.3.1 Identifying what works for you

Participants generated a broad range of seemingly novel behaviours that they managed, or later hoped, to engage with (see Table 2). While participants were not directed towards set topics for their goals, the BPS intervention encouraged participants to identify goals broadly categorized into physical activity, dietary or mental well‐being.

**Table 2:** Intervention‐generated goals (in the participant's own words)

Participant	Exercise behaviours	Dietary behaviours	Mental healthcare
2K	Climbing, yoga, running (including doing a 10K), walking 10,000 steps a day.	Bought more fruit and veg, prepared own lunches so as not to buy snacks/unhealthy meals.	
3J	Dancing, yoga, exercise.	Cooked more, cut down on takeouts and sugary drinks.	Meditation, doing things she enjoys.
4J	Going to the gym, lifting weights.	Aimed to hit calorie/food targets.	
5D	Working out at home with help from YouTube videos, cycling.	Improved intake of proteins (with food and powders), continue to exercise, which makes her crave fresh fruit and veg.	
7B	Go to the gym three times a week.	Make smarter choices with food, eat well for 6 out of 7 days a week.	Structure work/life balance. Rethink existing relationships and develop new and better ones in the future.
8E	Walks/hikes exercise regularly.	Aiming to eat a balanced amount of nutrients and vitamins.	Yoga, physical exercise as a way to improve mental health/reduce anxiety.
9C	Box jumps, circuit training.		Yoga. 9C also believes that improvements to physical health and a feeling of social connectedness will boost her mental health.
11E	Gym (including stepper/running machines), outside running.		
12S	Increase physical activity when at home (in Italy), spend less time sitting down, use stairs rather than elevators.	Snack at work to prevent hunger‐fuelled binges at home, eat more fruit and veg, eat more whole‐grain stuff and more legumes and lentils.	Get more (quality) sleep.
13V	Running, cycling, climbing, swimming, yoga, walk more. Bike instead of using public transport.	Eat vegetables, cook meals at home, follow a balanced diet, select only the best and most nutritious ingredients.	Continue to engage in physical activity and sports, which allow 13V to 'relax (her) mind'. Follow the principles of mindfulness, practice meditation.

To generate this list of goals, participants appeared to use the intervention first to consider their current health levels. They then identified barriers preventing them from engaging with desired behaviours before generating solutions to overcome them. This frequently meant developing ideas that made choosing positive health behaviours that were easier or more fun (as seen in the subtheme ‘A Sense of Interconnectedness’). Sometimes, this required an increase in planning and organization, and in the context of the intervention, this process aided actual implementation and behaviour change towards their identified BPS.

‘I took up activities that I enjoyed, and that did not feel so much like “exercise”, for example, I started to go climbing once a week as well as doing yoga and running on nice days’ (2K)

There was a sense of people using the intervention to ask serious questions about themselves and their behaviour. For one individual, it appeared that the intervention gave them ‘insight as to why I wanted to be this “version” of myself and why I thought it was the “best” version’ (6C). For others, the intervention caused them to re‐evaluate what behaviours had previously worked and what could work for them in the future.

#### 3.3.2 Alternative goals

For some participants, control meant considering a wide variety of goals. For example, an improved sense of security was a common goal (either by working on one's education, social networks or career prospects), though this might have been important as a way to set the foundation for which various physical health goals could then be achieved. It is also possible that these seemingly non‐health‐related goals helped achieve mental health goals along the way.

‘When I think of my best possible self, I imagine being fully happy and content in every aspect of life. The main ones being happy within my job, financially stable and happily in a relationship’ (14M)

Appearance‐related goals were also referenced frequently by participants. Sometimes, appearance was a way to ensure that physical health goals were achieved.

‘You could look at those pictures of yourself from years ago next to ones of you now and really see the improvement’ (4N)

For others, reflecting on how they thought about their appearance was a way to facilitate their health goals.

‘I decided to be more body positive and focus on health rather than weight loss’ (3J)

A significant number of participants imagined a physical version of themselves as a way to encourage physical health change in the hope that this would allow them to feel better about their appearance over time, in which case, improvements to health were a way to ‘improve’ (from the individual's perspective) their appearance.

‘Having worked hard towards my health goals, I'll be a size 12’ (9C)

‘I imagine being confident wearing dresses and summer clothes due to being confident in my body’ (11E)

#### 3.3.3 Technology as an aid

The intervention, especially the goal‐setting aspect, encouraged many participants to utilize technology to help implement their behaviour change and achieve and track their new identified BPS. Participants reporting using used alarms, videos, step‐trackers, calorie checkers and a range of mobile apps to help them monitor and implement their goal behaviours.

‘I started also checking my phone app regularly to track how much I was walking and then started to do a target of 10,000 steps a day’ (2K)

‘I started using a YouTube channel that provides very detailed programmes to work out. I found it useful as you are free to choose the length and level of exercises at every workout. Being very detailed, it feels like having a coach guide a personalised session’ (5D)

As one participant noted (4N), the important thing was to enter data and engage with technology honestly. Skipping tutorial videos or lying about food choices on a calorie counter, for example, might look impressive, but it would not benefit one's health.

#### 3.3.4 Positive feelings generated by the BPS intervention

Several participants discussed the emotions generated by engaging with the intervention. For many, achieving their goals provided feelings of happiness. Though there was an acknowledgement that goals take time to achieve, behaviours in particular ‘became easier each time!’ (2K). In at least one case, there was a discrepancy between their current and possible future selves (8E); participants reflected on feeling proud of themselves, even if they still had a long way to go.

‘You kept going to the gym, gradually being able to increase the weight you lifted, and that felt amazing’ (4N)

‘I found being able to talk about how I wanted to be in the future reminded me of my motivations, which are so easy to lose sight of in our busy day to day lives’ (6C)

## 4 DISCUSSION

This study aimed to qualitatively assess how participants engaged with and utilized the BPS intervention as a T2D prevention tool. All participants submitted evidence reporting their experiences and engagement with the BPS intervention. The examinations of these accounts have highlighted several nuanced ways in which individuals used the intervention to conceptualize their health, set goals and receive affective benefits, which may offer insight into how people can personalize simple interventions to meet their health needs.

To our knowledge, this is the first study to tailor a positive psychology intervention, and specifically the BPS intervention, for use as a public health application in the prevention of T2D. While we did not focus the BPS intervention on specific T2D risk factors for behaviour change (e.g., improvements in physical activity, smoking behaviour or diet), it is an important finding that participants incorporated these risk factors (at their choice) into their accounts. This intervention enabled people to seemingly person‐centre the intervention's focus and identify their best possible selves in the context of positive health behaviours and outcomes, highlighting an underpinning motivation to achieve good health and well‐being. We, therefore, propose that our tailored BPS intervention could be used as a flexible, brief and easily administered tool to assist public health efforts in encouraging people to change their behaviours for health improvement.

Our findings highlight how participants thought about and conceptualized their health using the BPS intervention. We reported that participants considered a broad definition of ‘health’ that encapsulated their physical, social and emotional needs. There was as much a need to address physical health behaviours and malaises, as there was to secure social relationships and make progress towards better mental health. In some cases, participants viewed these varying aspects of health as intertwined (as part of a larger whole), whereby physical health goals could only be met by working towards better mental health, and vice versa. Social goals were important for achieving mental health goals too, and there was evidence that social relationships also helped support physical health goals. This consideration of an individual's physical, social and mental health helped encourage several participants to reflect on the importance of their health journey. In framing it as such, these same individuals became aware of the role that patience and self‐forgiveness could play in achieving their long‐term goals. This is important, given that self‐forgiveness has been shown to be associated with reductions in self‐condemnation, which has consequences for physiological functioning,^69^ highlighting the reality of this interconnectedness and suggesting a potential BPS intervention mechanism that previous research has not considered (see Heekerens et al.,^70^ for a discussion around BPS mechanisms). Alternatively, these meditations may be evidence of self‐compassion (i.e., the ability to treat oneself with kindness, be nonjudgementally present and accept flaws as part of the human condition^71^), which has positive consequences for health and well‐being over time.^72^

We also explored how participants utilized the BPS intervention to control their imagined future and their current self. Participants used this tailored BPS intervention to seemingly gain a better sense of control over themselves and their health. Participants used the intervention to create a space where they could reflect upon their current selves, identify barriers to their future selves and develop thoughtful goals. As a result, participants appeared to move from goal identification to implementation, using additional resources, objective health markers and a sense of well‐being identified by engagement with the intervention. This is particularly noteworthy, given the gaps highlighted in the literature between setting health intentions and implementing said behaviours.^73^ This BPS intervention appeared to bridge the goal‐setting and implementation gap via these psychological changes in control, although affective changes brought about by the intervention may also have played a role.^73^

Regardless of how individuals considered and acted upon their identified BPS, participants made frequent references to positive emotions, especially as they set, made progress towards and achieved their various goals. Most of these feelings could be construed as evidence for the facilitation of positive affect (‘happy’, ‘proud’, ‘strong’, etc.^74^), but there was also evidence of gratitude. We also identified how the BPS intervention might have promoted motivation. Previous work has made connections between the BPS and motivation, with one set of authors claiming that just the act of ‘envisioning one's best possible self' is likely to be ‘inherently self‐relevant and motivating’.^34^ A qualitative investigation into BPS mechanisms^52^ found evidence that the BPS was at least partly consistent with the Self Determination Theory (SDT),^75^ a macro‐theory of human motivation. SDT proposes that people have three inherent needs that promote optimal motivation, development and wellness: autonomy (the sense that one's actions are under one's control), competence (the notion that one is capable and skilled) and relatedness (the feeling that one is close and connected to others).^75^, ^76^ There is evidence here that we have found something similar, given the importance of control (autonomy), self‐reported feelings of achievement when working towards or completing one's goals (competence) and social well‐being/relationships (relatedness) identified in participants' accounts. This is an important finding, given that motivation, self‐efficacy (i.e., notions of competence) and social factors have been highlighted as the key determinants of engaging in various health behaviours,^77^ further supporting the notion that the BPS intervention could be used to assist a range of targeted health behaviours.

In this context, we are reporting on the psychological and affective determinants of *individual* health behaviours, which we suggest are often missed from inclusion in the rollout of public health interventions, such as prevention for T2D intervention, which focuses purely on promoting behaviour change.^78^ Individual variation in the needs and determinants of behaviours fluctuate. Therefore, any public health interventions that aim to target large population groups must be flexibly aligned to individual determinants and support the behavioural and underpinning psychological and affective changes.

In the future, the BPS intervention could be utilized to enhance the quality and efficacy of public health interventions. The intervention could be made available as an online resource, integrated into existing public health interventions or provided in‐person by a healthcare professional. Those wishing to implement our tailored intervention for T2D prevention may utilize it as a brief psychological intervention in the same way that others have for addressing smoking^79^, ^80^ and drinking^81^ behaviours. If the BPS intervention was delivered face to face as part of ongoing consultation, healthcare professionals might ask recipients to share their goals with them to develop a health plan together. Alternatively, the BPS intervention could enter the health promotional field to enhance the ‘Make Every Contact Count’ (MECC)^82^ application; since healthcare professionals are familiar with this training already, they know about behaviour change, reflection and goal‐setting skills^83^ and this could be a new tool to help navigate the psychological changes required to assist with the behaviour changes associated with MECC interventions.

We proposed that an effective public health intervention should promote psychological and affective change to guide the uptake of healthy behaviours. Our tailored BPS intervention was designed to be a flexible and open intervention subject to participant choice in their application. While we aimed to consider this in the context of T2D prevention, the intervention could be applied to address a range of public health topics and promote specific health outcomes. Future work could also assess whether this version of the BPS is acceptable among other populations, especially since our sample was predominantly comprised of working‐age females. Therefore, further research exploring the application of this intervention in males may be advisable.

This study collected the post‐BPS intervention accounts from participants and analysed their experiences and reflections of its use and application. All participants submitted data; this alone may indicate its value to individuals and the acceptability of the intervention. However, while participants identified health goals and suggested behaviour change, we did not follow up measures of behaviour change (improvements in dietary, physical activity or well‐being), which are outcomes that warrant future quantitative research on the implementation of this intervention. To help our readers assess the transferability of the findings of this qualitative study to other contexts, we have provided clear methodological and analytical descriptions and offer verbatim quotes from the raw data to act as evidence to support our commentary.

## 5 CONCLUSIONS

Considering the risk that T2D and other preventable diseases pose to individuals (and the wider public health of the population), we must continue to provide opportunity, capability and motivation^84^ to manage their health by using novel, accessible and evidence‐based interventions. An investigation of this tailored BPS intervention has shown that, given space, participants engaging with this intervention may consider a holistic view of their health, identify barriers and facilitators to their best possible selves and develop tailored goals in a way that promotes positive affect, gratitude, novel thoughts and behaviours.

## CONFLICT OF INTERESTS

The authors declare that there are no conflict of interests.

## ETHICS STATEMENT

Ethical approval for this study was obtained from the Liverpool John Moores University Research Ethics Committee, and participants provided full consent to participate in the study and consented to verbatim extracts of their transcriptions being published.

## AUTHOR CONTRIBUTIONS

*Conceptualization, methodology, validation, formal analysis, investigation, data curation, writing original, writing*—*review and editing, visualization*: Ben Gibson. *Conceptualization, validation, formal analysis, writing—review & editing, supervision, project administration*: Kanayo Umeh. *Conceptualization, recruitment of participant strategy, writing‐review & editing, supervision support*: Ian Davies. *Conceptualization, methodology, validation, formal analysis, resources, data curation, writing—original, writing‐review and editing, visualization, supervision*: Lisa Newson.

## Data Availability

Raw data have been included as evidence via extracted quotes from verbatim transcripts as samples of evidence. Full transcript release has not received ethical approval or participant consent. For further study details, please contact the corresponding authors. The authors confirm that the data supporting the findings of this study are available within the article.
